# DS-PACK: Tool assembly for the end-to-end support of controlled access human data sharing

**DOI:** 10.1038/s41597-024-03326-9

**Published:** 2024-05-15

**Authors:** Pinar Alper, Vilém Dĕd, Sascha Herzinger, Valentin Grouès, Sarah Peter, Jacek Lebioda, Linda Ebermann, Marina Popleteeva, Nene Djenaba Barry, Danielle Welter, Soumyabrata Ghosh, Regina Becker, Reinhard Schneider, Wei Gu, Christophe Trefois, Venkata Satagopam

**Affiliations:** 1 Luxembourg National Data Service, PNED GIE, Esch-sur-Alzette, L-4362 Luxembourg; 2 ELIXIR Luxembourg, Belvaux, Luxembourg; 3 Luxembourg Centre for Systems Biomedicine, University of Luxembourg, Belvaux, L-4367 Luxembourg

**Keywords:** Data publication and archiving, Software, Standards

## Abstract

The EU General Data Protection Regulation (GDPR) requirements have prompted a shift from centralised controlled access genome-phenome archives to federated models for sharing sensitive human data. In a data-sharing federation, a central node facilitates data discovery; meanwhile, distributed nodes are responsible for handling data access requests, concluding agreements with data users and providing secure access to the data. Research institutions that want to become part of such federations often lack the resources to set up the required controlled access processes. The DS-PACK tool assembly is a reusable, open-source middleware solution that semi-automates controlled access processes end-to-end, from data submission to access. Data protection principles are engraved into all components of the DS-PACK assembly. DS-PACK centralises access control management and distributes access control enforcement with support for data access via cloud-based applications. DS-PACK is in production use at the ELIXIR Luxembourg data hosting platform, combined with an operational model including legal facilitation and data stewardship.

## Introduction

Clinical and translational research relies on the use of biomedical data collected from human subjects, often called “human data”. Human data differs from other research data due to its sensitivity and personal nature. Collecting, handling and sharing human data requires preserving subject privacy and data confidentiality. The research community has developed the so-called “controlled access” model for data sharing to address these concerns^1^. In this model, a researcher who wants to re-use human data from prior studies needs to make a formal access request to data controllers. The request is reviewed by a data access committee (DAC), composed typically of investigators responsible for the primary data/sample collection. The DAC review provides the necessary oversight and authorisation, ensuring the proposed reuse of data honours the use conditions placed by data donors and meets the necessary ethical and legal requirements. It is the responsibility of the researchers who produce data and would like to share it to ensure that the necessary operational procedures are in place so that the data is ingested into the controlled access realm, is findable through well-defined metadata, and is accessible via documented auditable processes.

Putting in place controlled access for one or more datasets is a complex and resource-intensive undertaking often performed by specialist intermediaries rather than researchers themselves^2^. The most common way for researchers to provide a controlled access layer over research data is either by depositing the data into centralised human genome/phenome archives^3,4^, or by handing data over to a “data support” team so that that team becomes the contact point for data access requests and handles the downstream processes. In both models, these intermediaries perform some or all of data discovery, access request management, data storage and delivery functions.

In recent years, several developments have diminished the choice of centralised repositories. First, the European General Data Protection Regulation (GDPR)^5^ has restricted the cross-border movement of data as it requires additional safeguards during data collection and sharing^6,7^. Researchers now need to consider complex ethico-legal aspects before depositing data to archives outside the country of data collection and/or the EU, as obtaining necessary safeguards may be too time-consuming or, in some cases, impossible. To accommodate GDPR requirements, repositories have proposed “federated” data sharing models^8^. In a repository federation, the data discovery function remains centralised; meanwhile, data storage and delivery are handled by distributed, often national, nodes. The emerging federated approach succeeds in keeping data within national borders; however, it currently has two functional gaps. First, managing access requests is left to the nodes for them to coordinate – among requestors, providers and access committees – the review of the requests and the conclusion of respective data use agreements.

Second is the evolution in modalities of data access and analysis. The traditional mode of data access has been file downloads by authorised requestors. We are now seeing – partly due to the GDPR – an increase in the adoption of the so-called “compute to data” approach, where being granted access to a dataset means being able to access and run analysis in a cloud environment that contains the dataset rather than being able to download the data. Current federation implementations support data access via file downloads, for clinical and translational data in particular; this approach prevents the incorporation of various data-sharing cloud platforms into federations^9^.

The landscape changes initiated by the GDPR have become the impetus behind institutional efforts to establish local data support teams and controlled-access processes. The challenge that awaits these teams is (1) a lack of software tools that would support implementing the controlled access process, (2) a lack of formalised roles for the ownership and improvement of the process^10^ and (3) the necessity to address GDPR accountability requirements^11^, such as audit trails and documentation, without dragging the whole process.

As a member of ELIXIR^12^, a pan-European life science information infrastructure, ELIXIR Luxembourg (https://elixir-luxembourg.org) is the data support partner in several European projects. Over the years, we have had to overcome the said challenges. As of today, ELIXIR Luxembourg hosts a data catalog^13^ and provides controlled access to diverse types of human datasets in various modalities, either via file transfer or in cloud environments and applications, whereby data is brought to life through curation, integration and analytics. The solution blueprint that underlies our controlled-access setup is the Data Stewardship Provenance and Compliance Kit (DS-PACK). DS-PACK is an assembly of open-source tools that:

- acts as middleware translating the information from the data access request management system to commonly used authentication and authorisation protocols, thereby allowing the sharing of data via any hosting platform supporting these protocols;
- solves a problem inherent in distributed and federated data sharing, which is the centralisation of access control over distributed, heterogeneous hosting platforms;
- supports the sharing of diverse biomedical data types and data delivery in different modalities;
- semi-automates the controlled access processes by outlining clear roles for data support teams, which can relieve pressure from DACs and help scale up data sharing;

In this paper, we provide an overview of DS-PACK and illustrate how it has been put to production use within ELIXIR Luxembourg’s data hosting service. We outline the three core contributions of DS-PACK as (1) a standards-compliant access control pattern implementation for research data platforms, (2) an open-source and reusable component assembly, and (3) an implementation of the data protection by design and by default (DPbDD) principle of the GDPR. We present the method adopted to build DS-PACK. Finally, we review related work and discuss how DS-PACK relates to existing approaches and discuss our future work.

## Results

### Centralised access control for distributed, heterogeneous data hosting platforms

When data is shared over distributed platforms, an inherent problem is managing access control lists (ACLs) for each platform. List management becomes a bottleneck as the number of platforms and granted accesses increase. DS-PACK adopts an existing information security pattern to address this issue, where the ACL is maintained centrally and necessary information is propagated to hosting platforms during data access.

**Fig. 1:**
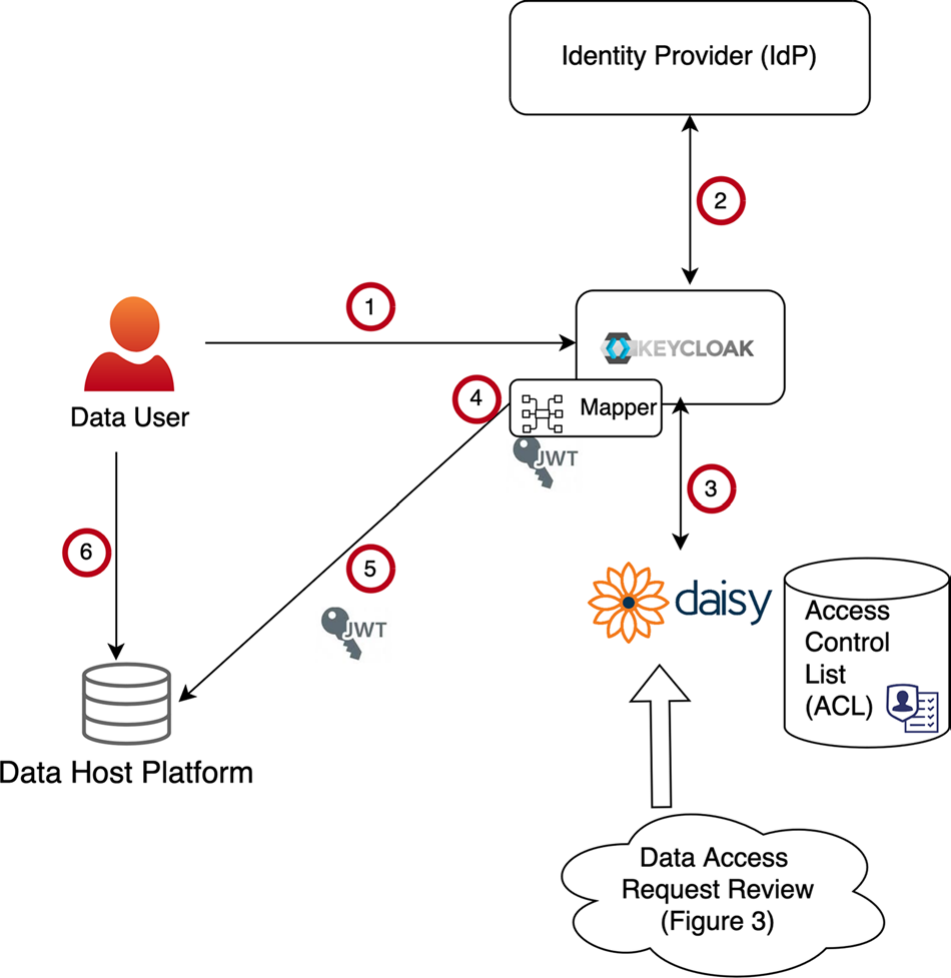
Authentication and access control in DS-PACK. (1) User is directed by the data host platform to Keycloak for authentication. (2) User is authenticated via their associated identity provider. (3) Keycloak pulls the user’s permissions from the access control list in DAISY. (4) Using mappers for the data host platform the permission information is packed into JWT tokens. (5) The user identity and permissions are presented to the data host platform in JWT tokens. (6) If permitted, the user can access data on the platform.

Figure 1 illustrates ACL management in DS-PACK, and the next section describes its software components. We use the Data Information System (DAISY)^11^ to store and maintain the central ACL. Access information is a triple, which is the combination of (1) the user having access, (2) the persistent identifier (PID) for the dataset given access, and (3) the provenance of the access record, including time of creation and the record’s source. Accesses are created following the access request review.

DS-PACK uses the Open ID Connect (OIDC) standard^14^ to authenticate the user and and it uses JSON Web Tokens (JWT)^15^ to transport authenticated user identity and permissions. We use Keycloak (https://www.keycloak.org) as the identity and single sign on platform. Keycloak supports external identity providers (IdP); user authentication can be performed in a federated manner, allowing users to log in with an existing account in any other trusted IdP that supports the OIDC standard, e.g. user’s home institution, ORCID or Life Science AAI Login (https://elixir-europe.org/platforms/compute/aai). Any information the IdP provides can also be collected and used for internal user management, e.g. utilising user affiliation information in access request handling and review.

DS-PACK includes mappers - one per hosting platform - that pack authorisation information into JWT tokens so the recipient platform can consume it without code changes. Transporting authorisation information in user tokens is an efficient and adopted pattern in information security. Such a solution allows authorisation to be propagated only to the platforms the user accesses without custom propagation logic.

### Open-source tool assembly for the end-to-end support of the controlled access process

**Fig. 2:**
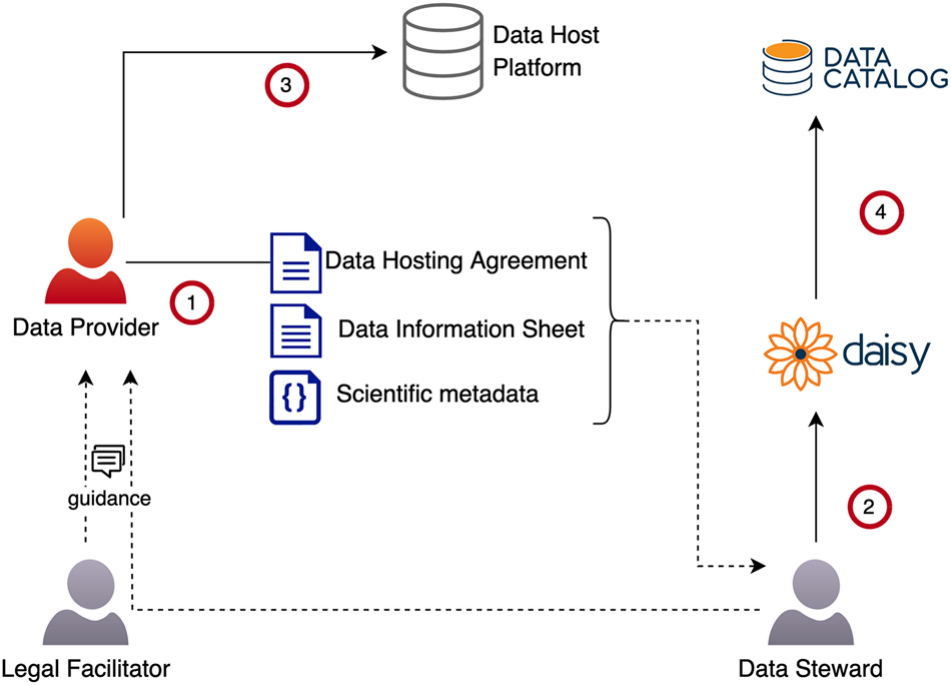
ELIXIR Luxembourg data submission process based on DS-PACK. (1) The data provider customises the data hosting agreement template to suit their requirements with guidance from the legal facilitator, provides access policy and ELSI, and GDPR metadata by filling out the Data Information Sheet (DISH) with guidance from the data steward, and provides scientific metadata for the dataset in DATS JSON format. (2) The data steward imports the DISH and the DATS JSON into DAISY to create the corresponding dataset record(s); the data hosting agreement is attached. (3) The data provider makes the data available on a host platform maintained either by the provider or by ELIXIR Luxembourg. (4) The data steward finalises the dataset record by adding information on data (host) locations, and publishes the dataset; as a result, the dataset gets a persistent identifier and becomes visible in the Data Catalog.

**Fig. 3:**
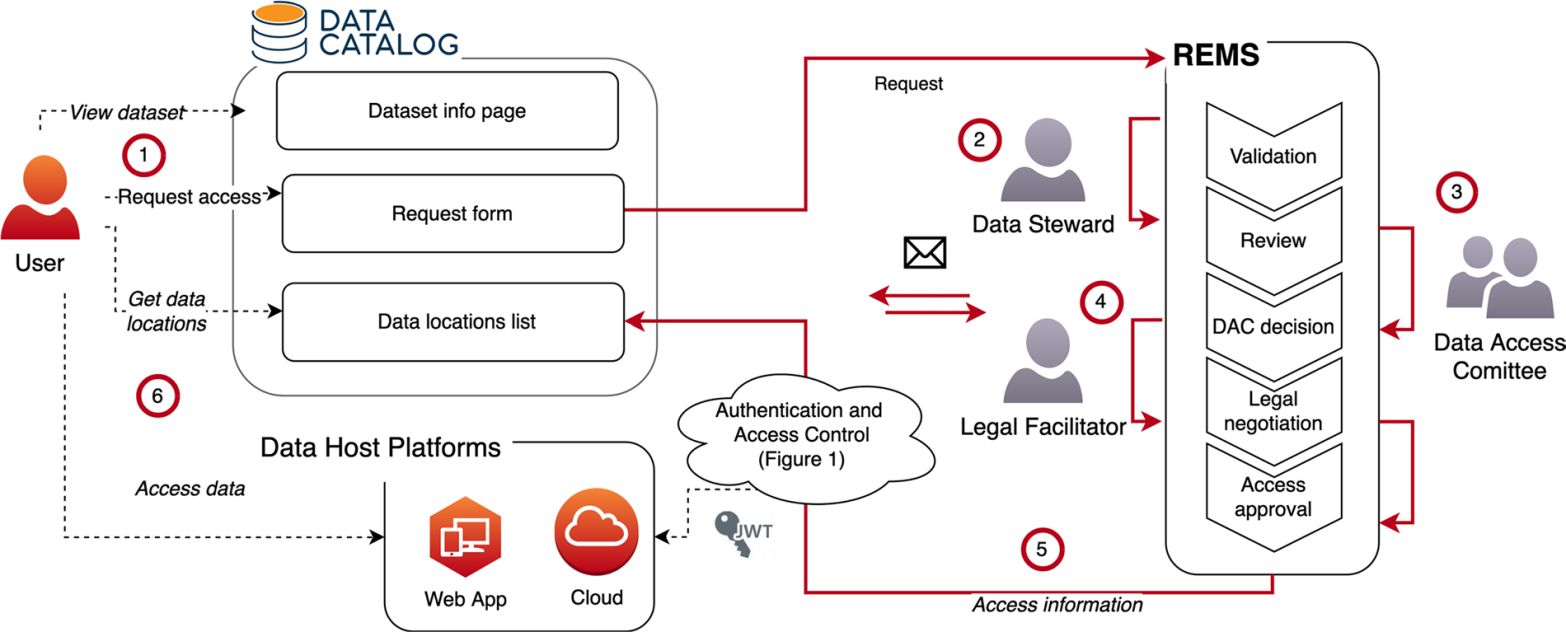
ELIXIR Luxembourg data access process based on DS-PACK. (1) The user locates the dataset in the catalogue and initiates an access request filling out the form associated with the dataset. (2) The data steward reviews the request and forwards it to the DAC. Additional information can be received from user via email. (3) The DAC reviews the request and makes a decision. During the review, the DAC may ask the data steward for further information. (4) Following a positive decision, the user customises the data use agreement template with guidance provided by the legal facilitator. (5) When the data use agreement is signed, the decision result is propagated from REMS to the access-control master in DAISY. (6) The user accesses the data in the data host platform. For each access attempt, Keycloak will consult the access control list in DAISY and present the user’s permissions to the host platform in their access token.

The DS-PACK aims to provide automation support for the controlled access process. We achieve this with an integrated assembly of software tools, metadata collection and agreement templates; we also provide a recommended operational model and identify roles for intermediaries similar to ELIXIR Luxembourg. DS-PACK is based entirely on open-source tools and templates, which implement standards where applicable. Component descriptions are given in following subsections, and the assembly’s operation for ELIXIR Luxembourg’s data submission and access process are given in Figs. 2, 3 (user authentication is omitted from these figures, as it is discussed in the previous section).

The DS-PACK operational model provides end-to-end data submission and access support, including legal facilitation and data stewardship. These two activities, which are further described in this section, make up the two main manual parts of the process. ELIXIR Luxembourg concludes agreements with providers and users separately, removing the need for these parties to enter legal processes in a peer-to-peer fashion. We provide templates for data hosting and data use agreements, which can be used as-is or with minimal customisations by legal facilitators. The combination of templates and facilitators brings efficiency to the legal processes and ensures that the necessary provisions concerning protecting sensitive human data are in place.

The data providers submit ELSI metadata for the datasets shared under controlled access, including use conditions, in data information sheets (DISH). They also provide additional structured metadata about the content of the datasets and the modalities of the studies from which they were generated. Specifically, metadata describes (1) the datasets that are shared under controlled-access, (2) the data sources, specifically the cohort studies in which the clinical and translational datasets were collected, (3) the data providers, namely the research project and principal investigators that are making the data available for re-use. Details of the schemas and ontologies utilised for metadata are described in the next section under the“Data Catalog”.

Data stewards import ELSI and scientific metadata into DAISY to obtain draft metadata records. ELSI metadata is further curated and verified by reviewing the provider’s access policy and access agreement documents. Once finalised, data stewards publish the metadata in the data catalog, making datasets discoverable by prospective data users.

Data stewards support the access process by validating the identity and affiliation of the requestors and by an initial assessment of their eligibility to access data based on defined use conditions. Requestor registration and identity checks are typical in secondary use of human genome/phenome data and have been formalised as “registered-access”^16^. The identity checks performed by data stewards are intended to offload such duties from the data access committee so that the committee can focus checking whether the proposed secondary use of data in the requestor’s research project is inline with the purposes for which the data is collected.

Data stewards also mediate the communication between requestors, the data access committee (DAC) and the legal facilitator, maintaining frequently asked questions on datasets and collecting from the requestor any further information required by the DAC.

#### DS-PACK Components: software tools

**Data Information System (DAISY)**^11^ is a dataset registry and documentation tool meeting GDPR accountability requirements for biomedical research projects. DAISY is targeted for the use of data stewardship teams and is central to the operation of DS-PACK, acting as ELSI metadata and access control master.

**Keycloak** (https://www.keycloak.org) is a platform for single sign-on with identity and authorisation management. It implements the Open ID Connect (OIDC) protocol^14^, which uses signed and verifiable JSON Web Tokens^15^ to transmit user authentication and authorisation information to clients.

**Data Catalog**^13^ is a dataset information index and search tool based on the DAta Tag Suite (DATS) schema^17^, which is fully interoperable with the W3C Data Catalog Vocabulary (DCAT)^18^. Discipline-specific metadata is then added to catalog records in the form of key-value pairs, thanks to the flexibility of the DATS model. A range of ontologies are used in the Data Catalog, including but not limited to the Data Use Ontology (DUO)^19^, the Semanticscience Integrated Ontology (SIO)^20^, the National Cancer Institute’s Thesaurus (NCIt)^21^, the MONDO Disease Ontology^22^, the EDAM Bionformatics Ontology^23^, the Units Ontology (UO)^24^ and the CHEBI Chemistry Ontology^25^.

**Resource Entitlement Management System (REMS)**^26^ is a tool for creating and executing workflows assessing data access requests. Applicants can utilise their federated user IDs to access REMS, complete the data access application, and acknowledge the use conditions associated with the resource. Once the application has been submitted, REMS assigns it to the workflow handler, e.g. a data steward or a data access committee member, for review and approval. Additionally, REMS can generate reports detailing the status of the applications and the data access rights that have been granted.

**Data Host Platform** is any platform that implements the OIDC standard and consumes authorisation information from JWT tokens. Currently, we have integrated the ADA Platform (https://ada-discovery.github.io) into DS-PACK, and we are working on integrating RedCAP^27^. Host platforms have to implement de-serialization and resolution to platform-specific access permissions. Timely exchange of authentication and authorisation information with Keycloak, i.e. upon each login and token expiry, is also a responsibility of the platform.

#### DS-PACK Components: templates

**Data information sheet (DISH)**^28^ is a metadata collection instrument, organised as a questionnaire spreadsheet, to capture information on Ethical Legal and Societal Issues (ELSI) concerning the re-use of data. We refer to this information as “ELSI Metadata”^29^, primarily composed of data use conditions stemming from consent clauses, data provider policies and access agreement conditions.

**Data hosting agreement**^30^ is a legal document signed between the data host and the data provider(s) that contains the provisions for long-term data hosting, GDPR-compliant data access to third parties, as well as additional data management services, where needed, such as data curation or (re)pseudonymisation. The provider signs the data hosting agreement and, in the annexe, provides the DISH. The Data Access Policy is built upon the information provided by the Data Provider through the DISH.

**Data use agreement**^31^ is a legal document signed between ELIXIR-LU and the data user’s institution that contains the provisions for the GDPR-compliant use of data, including use purpose limitations, storage durations, non-transferability, right of data subjects and information security safeguards.

**Data user responsibilities acknowledgement**^32^ is a declaration which accompanies the data use agreement; it is signed by each data user that is listed as a prospective data accessor in the access request form; users confirm they will comply with the data use conditions and general good scientific practice for data handling.

### An implementation of data protection by design and by default

Data protection by design and by default (DPbDD) is a provision of the GDPR^5^ requiring data controllers to think ahead on data protection and incorporate appropriate technical and organisational measures into the design of data processing systems. DS-PACK assembly follows DPbDD through its four key features: ELSI Metadata, DAC Review, Centralised ACL management and model agreements. We discuss, next, how each DS-PACK component supports GDPR’s data protection principles through these features. A mapping of components to principles is also given in Table 1.

**Table 1:** GDPR data protection principles supported by the DS-PACK components.

	DISH	DAISY	Keycloak	Data Catalog	REMS	Data Host Platform	Agreements
Transparency, Lawfulness, Fairness	x	x			x		x
Purpose limitation	x	x		x	x		x
Data minimisation	x	x	x		x		x
Storage limitation		x				x	x
Confidentiality and integrity		x	x			x	x
Accountability		x	x	x	x	x	x

#### Transparency, lawfulness, fairness

ELSI metadata captured in DISH and recorded in DAISY requires the data providers to declare the legal bases for data collection, sharing and secondary use, respectively. Using REMS, the DAC verifies whether the proposed use conforms to data use conditions originating from informed consent, contributing to transparency and fairness of secondary use of personal data. The data agreements, both the data hosting agreement and the data use agreement, oblige the data host/repository, the data provider and the data user to comply with Article 5 of the GDPR, which lays out the core data protection principles listed in Table 1.

#### Purpose limitation

**Fig. 4:**
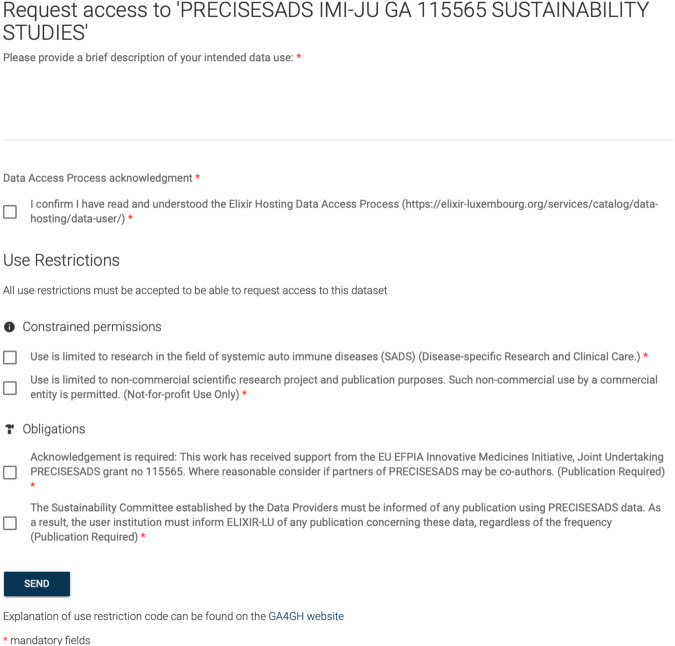
An example access request form. The user is presented with all data use conditions and the required data access agreement. Best practices for studies collecting human data recommend consent with “broadly described research purposes with ongoing updates for participants” as well as allowing “participants to retain control”^44^. The “Disease-Specific Research”^19,45^ use condition illustrated in this screenshot is common in controlled access human data sharing and it emerges from the so-called “Tiered-Consent” model^46^. This model allows study participants to retain more control over their data by giving them the option to consent to data uses in particular research categories or settings. Compared to broad consent, the tiered model can be seen as a compromise allowing participants to opt in to share their data, which otherwise would be confined to the primary study and not shared.

A crucial part of ELSI metadata is data use conditions, which outline the permitted and prohibited uses of data and the obligations that shall be fulfilled before access. Respecting conditions that descend from participant informed consent is a primary responsibility for data sharing under the GDPR^33,34^. Figure 4 displays use conditions originating from informed consent, such as disease-specific research use, and conditions commonly required by data providers, such as ethics review or collaboration requirements.

Publishing use conditions in the data catalog ensures that prospective data users are informed when requesting access. The DAC review of the proposed data use then verifies conformance to data use conditions. The data hosting agreement holds providers liable for providing metadata that allows GDPR-compliant processing of the data by the host/repository and the data users; meanwhile, the data use agreement restricts the data user’s use of the data to the DAC-approved purpose.

#### Data minimisation

providers are asked to confirm that the data is pseudonymous and contains no standard personal data attributes as part of the ELSI metadata declaration. The DAC also reviews whether the proposed research requires all variables of the data or whether a data subset needs to be requested. Accesses granted are contractually time-bound for a default period of one year; after this, researchers need to renew their access request and the data use agreement.

#### Storage limitation

For datasets hosted in the repository, DAISY acts as the central register of data storage locations and data hosting platform endpoint URLs. The data hosting agreement mandates limited durations of data storage by data users, which are tracked in DAISY. Data stewards get notified of datasets nearing the end of their storage period. In addition, by allowing cloud data-sharing platforms that access without data download, the DS-PACK eliminates the need for storage limits tracking with the users of such platforms. To support research reproducibility, the data hosting agreement foresees long data retention periods for the host repository. The agreement is concluded typically for a minimum of 10 years, and it contains provisions for an additional data retention period of 15 years upon agreement termination.

#### Confidentiality and integrity

Ensuring confidentiality is the primary goal of DS-PACK. Researchers can access only those datasets for which they have been granted permission. Accesses are centrally managed and globally enforced, a design pattern implemented with DAISY, Keycloak and data platform integration. Upon a positive DAC response, access expiration is set by the workflow handler in REMS and propagated to DAISY. Upon completion, the access requires an application for renewal and a new DAC review. DS-PACK adopts conservative login policies; user tokens are short-lived, and logins to a platform are only allowed when at least one permitted dataset exists on that platform. Confidentiality obligations are standard components of all agreements. In particular the data use agreement outlines responsibilities for the user’s host organisation in cases of misconduct such as attempting to re-identify data subjects or compromise subject identity. Data stewards can manually revoke access in case of misconduct or when the users leave the institution before the end of the access expiry period.

DS-PACK handles and grants access per named user and does not support user groups. Individual data hosting platforms handle data confidentiality during storage, analysis and transfer. There is no technical barrier to having user groups and assign permissions to those. We observe, however, that having groups adds a level of indirection to the representation of ACLs, as user permission is no longer an explicit record but would need to be deduced from group membership. This indirection bears potential for obfuscating the audit trail of accesses obtained and lost by users. In addition, our agreements list named users and we see value in user participation in the access request process to raise awareness of data use conditions and user responsibilities.

#### Accountability

DS-PACK addresses the accountability requirement by documenting sensitive datasets, accesses and the provenance of access decisions. DAISY is the central register of sensitive data and the logbook of activities concerning data, thereby implementing the “Register Of Processing Activities” (ROPA) outlined in the GDPR. The entire process, from access request to DAC review, the population of ACL lists and the enforcement of access are automatically logged. Logs are easily correlatable by user names and dataset persistent identifiers (PID). The audit trail for the DAC process is recorded in REMS, and individual accesses are logged in Keycloak and the hosting platforms. Any manual updates to ACL lists in DAISY such revocations are logged.

Finally, we have performed a Data Protection Impact Assessment (DPIA) for the ELIXIR Luxembourg data hosting setup based on DS-PACK. The DPIA outlines privacy risks and mitigations, thereby providing evidence of the decision-making behind the DS-PACK design. Our model agreements ensure that involved parties follow all principles and clearly define their accountability. There are differences in GDPR interpretation by the national data protection authorities and consequently differences in national implementations. Our agreement templates are GDPR-observant and general-purpose. In case they are to be used in countries with distinct national GDPR provisions, then those would need to be reflected to the agreements.

## Discussion

### Related work

The US-based dbGaP^4^ is one of the oldest deposition databases for subject-level genome/phenome data; as such, it has set a precedent for the controlled access model and various other data sharing initiatives^35^. dbGaP requires data submitters to delineate all data use conditions as data use limitation tags on datasets. To streamline data access, dbGaP concludes an online Data Use Certification Agreement digitally confirmed by the data requestor and a representative from their institution. Data provision is through time-limited file downloads, and dbGaP notifies data users to delete local copies after one year or to renew their access.

EGA^3^ is a controlled access repository of the European Molecular Biology Laboratory and the Center for Genomic Regulation. EGA provides partial support for the controlled access process focusing on dataset discovery and provision, leaving out the DAC orchestration and the agreement facilitation. EGA users are provided with the contact details of DACs associated with datasets from whom they can request access. As guidance to DACs, an access agreement template is provided; each DAC, however, is responsible for drafting their agreement template and concluding individual agreements with requestors.

The EGA is developing a “federated” model (FEGA), where data discovery occurs via the central repository and the data hosting and provision is done by (local) nodes^8^. Like the dbGaP, EGA and FEGA data provision is also based on file downloads. A federated sharing model is arguably more complex than a centralised one. Despite its complexity, the federated model has emerged as a response to the GDPR. Particularly GDPR-required safeguards for cross-border data transfer and a lack of clarity and shared interpretation at the EU level^36^ make it difficult for researchers to deposit data to central repositories. E.g. data collected from a Luxembourgish cohort without consent provisions for sharing outside the country cannot be transferred to centralised repositories. An advantage of federations, which keep the data close to source is that each node can continue to complete and curate the data without the need to transfer different releases to a central repository and instead transfer its metadata only.

GA4GH Passports^2^ is an open standard that focuses on transporting a user’s access rights, called visas, along with the user’s identity, called a Passport. The standard outlines visa types, representing the typical information that must be transported when researchers access cloud environments holding open or controlled access genomics and health datasets. Examples of visas are a researcher’s institutional affiliation, role, other linked identities and finally, the datasets to which they have been granted access. The Passports standard is based on the OIDC standard; as such, it uses digitally signed JWT tokens that ensure the identity and access information in tokens are authentic and verifiable. GA4GH Passports is a recent standard for which few demonstrators have already implemented, including the test implementations for the dbGaP and the EGA repositories.

Sage Synapse^37^ and the Open Science Framework^38^ are two research collaboration and results sharing platforms that promote Open Science practices. For sensitive human data, these platforms offer the “controlled access” option. The Synapse platform requires data contributors to designate data use conditions. The platform facilitates an access request process directly between the data requestor and the contributor not utilising DACs or data sure agreements. The OSF, on the other hand, achieves controlled access by directing its users to designated repositories.

### DS-PACK present and future

DS-PACK has been in production use at ELIXIR Luxembourg since 2021 to deliver our node’s data hosting service. We have coupled DS-PACK with an operational model, where legal facilitators and data stewards act as intermediaries by tailoring agreements, facilitating DAC-user communication, data request validation and ensuring ELSI metadata precision. In this regard, our solution represents a middle ground between those repositories with highly streamlined access procedures and fixed agreements, such as the dbGaP, and others that leave out support for legal facilitation and DAC orchestration such as the EGA. DS-PACK operational process involves human elements, which raises the question of scalability. Centralised repositories such as the dbGaP and the EGA have succeeded in vertically scaling data sharing with large numbers of datasets and access requests. Now the emerging federated models call for a horizontal scaling of data sharing. The goal of DS-PACK is to be part of that horizontal scaling by empowering institutional data support teams and to establish data sharing nodes in federations.

Data support teams are lean; they undertake diverse responsibilities with limited human resources. Practical duties of ELIXIR Luxembourg data stewards involve responding to inquiries from data submitters and requestors, facilitating communication, curating ELSI metadata, and ACL list management. List management is not as time-consuming as other tasks, but it requires high accuracy in implementation otherwise leading to non-compliance. The automation-support that the DS-PACK brings both time savings, as in the case of REMS facilitating communication of various parties and increased accuracy as in the case of centralized ACL management.

Any system that supports OIDC-based authentication and authorization, can be connected as a data host platform to DS-PACK. The effort required for integration ranges within a few days of development or configuration work. A natural next step for us is to implement support for the GA4GH Passports specification and enable our “mappers” to generate Passport-compliant tokens from ACL lists. The use case for Passports emerges for data users navigating environments with different trust levels, such as when the user needs to access data in a cross-organisational research data cloud environment.

Throughout the various multi-party clinical and translational studies we were involved, we observed that data sharing starts as early as the data collection, and the audience with which the data is shared changes over time. During the study, the data is shared within the consortium for primary use, and it is subject to the study’s ethico-legal framework, a model referred to as “clique sharing”^39^. Upon study completion, the data is shared with the broader research community for secondary use, which may require different legal provisions. The DS-PACK operational model allows us to deploy the same tool assembly for both primary and secondary use, and we will continue to deploy it for various consortium studies. The loose coupling of the DS-PACK components allow us to deploy the assembly partially depending on projects' requirements.

The DS-PACK design emphasises the collection and curation of ELSI metadata by data stewards. Metadata meets the documentation requirements of the GDPR as it contains all attributes identified by Article 30, and more. This information provides a baseline to build the necessary technical and operational safeguards towards GDPR principles. Our way of modelling use conditions allows us to specify in a more granular, therefore more precise manner which data uses are permitted and prohibited; we can also incorporate terms from different vocabularies such as the Data Use Ontology (DUO)^19^. To facilitate indexing of our data catalog by other thematic catalogs, we are planning to expose our DATS-based catalog content in DCAT form.

ELIXIR Luxembourg will soon join one or more controlled access data sharing federations at the EU level^8,40,41^. The DS-PACK development constituted our groundwork as middleware allowing us to establish our node repositories and operations in different federations. DS-PACK assembly is openly available for the use of research data support teams establishing thematic and/or institutional repositories or nodes within repository federations. All component software licenses allow commercial use; only the Data Catalog and DAISY require derivative software to stay open sourced. We are building easier containerised deployments and improving our documentation to assist adopters.

## Method

ELIXIR Luxembourg already had a process to support controlled access for project consortia. Our process was largely manual, using email communication and document-based forms. DS-PACK development commenced around the time the GDPR came into effect. We started by mapping out the existing process and identifying process gaps and priorities for automation; we also identified opportunities for metadata standardisation. In parallel, an ELSI expert at our node translated the GDPR principles and other relevant legal requirements into prospective technical and operational measures that could be adopted.

Before the implementation, we reviewed existing open-source tools to assess whether they could be used in our solution. The implementation of DS-PACK was iterative and incremental; automation was gradually introduced. In the first phase, we deployed DAISY to build our internal inventory of sensitive datasets, including ELSI metadata. Next, we introduced the Data Catalog into the assembly, exposing dataset metadata in a standard and findable manner. Finally, we added REMS, Keycloak and hosting platforms with mappers to automate DAC review orchestration and enforce access decisions.

DAISY utilised GA4GH’s Consent Codes, which are atomic data use terms representing common secondary use conditions for research data. We extended DAISY to store use conditions as triples, combining a use term with a use rule denoting whether the cited use term denotes permission, a prohibition or an obligation^42^. We added the ability to plug external PID generation services, e.g. DOIs, to support other PID types in addition to DAISY’s default internal accessioning scheme. Upon dataset publishing, the user must select the publishing target (catalog) and an existing REMS form. To support ACL management, we extended the DAISY information model and integrations. DAISY already provided a data logbook allowing data controllers to record data lifecycle events, including accesses. We refined the access schema to accommodate the ACL triple.

We connected the Data Catalog to DAISY’s REST endpoint to pull dataset descriptions, ELSI and discipline-specific metadata in DATS JSON format. This connection allowed the two systems to have synched information in near-real-time. We also extended the Data Catalog to act as a front end to REMS for the creation of access requests for selected datasets. The DAC review is triggered from the corresponding user form added to the data catalog interface. REMS is used as-is in DS-PACK, not requiring custom extensions. We configured the REMS to notify DAISY in case of granted accesses.

As Keycloak implements the OIDC standard, most of the custom work was limited to creating mappers for the JWT tokens that carry user permissions. For client applications hosting multiple datasets, the client must be capable of reading claims inside the JWT tokens and mapping them to certain local permissions. While this is technically relatively trivial, many applications added OIDC only rudimentarily to their historically already existing authentication solution. Fortunately, OIDC is growing in popularity, so adoption and familiarity with the protocol are improving, and with them, the level of integration. In simple situations where one application hosts one dataset, the only requirement for the integration into our access pipeline is the support of OIDC for authentication. The JWT tokens we use are signed, and thus immutable, and sent over an encrypted connection (HTTPS), and therefore not readable by anyone but source and target. We have, however, not encrypted the token content as the content is not sensitive in-of-itself and therefore its encryption would not add any value to our solution.

To facilitate ACL lookup from DAISY, we developed a plugin that extends the Keycloak authentication flow with a call to DAISY’s REST API when a given user logs in. By not relying on scheduled synchronisations but extracting this access information on-demand, we can ensure that the information is up-to-date when served to the client applications. We also added functionality to Keycloak that allows us to control access to an application at the authentication stage based on self-defined policies. In other words, we can prohibit users from logging into an application if they do not have a certain dataset access record. This allows us to integrate applications into our access control pipeline, even if the application only supports central authentication via OIDC but handles authorisation locally.

## Data Availability

The ELIXIR Luxembourg data catalog(https://datacatalog.elixir-luxembourg.org) currently lists 261 datasets; 27 of those are hosted in platforms managed by our node. Human datasets that are not of an anonymous nature fall under the GDPR, and therefore, they are available under a controlled-access regime such as the “PRECISESADS IMI-JU GA 115565 Sustainability Studies” dataset^43^, which is used for the testing of the DS-PACK assembly. The process for requesting a controlled access dataset can be initiated by clicking the “Request data access” button on the dataset information page in the catalog. Anonymous human datasets or, other, non-human data is available under open access via direct download by clicking the “Access data” button on the dataset information page.

## Code availability

Source code for the components of the DS-PACK assembly can be found in the following repositories. Among these, ELIXIR Luxembourg is the main developer of DAISY, Data Catalog and ADA systems and the Keycloak DAISY synch plugin.

• Data Information System DAISY, GNU Affero General Public License (AGPL 3.0), https://github.com/elixir-luxembourg/daisy

• Data Catalog, GNU Affero General Public License (AGPL 3.0) https://github.com/FAIRplus/imi-data-catalogue

• Keycloak, Apache 2.0 License, https://github.com/keycloak/keycloak

• Keycloak - DAISY synch plugin, Apache 2.0 License, https://gitlab.lcsb.uni.lu/keycloak-projects/keycloak-providers

• Resource Entitlement Management System (REMS), MIT License, https://github.com/CSCfi/rems

• ADA Discovery Analytics (ADA), Apache 2.0 License, https://github.com/ada-discovery

The source code to connect DS-PACK components is located within each component. We are working on a containerised demo of DS-PACK execution, which will be made available under the ELIXIR Luxembourg GitHub repository. https://github.com/elixir-luxembourg/
